# James W. Alexander, D.D.

**Published:** 1866-11

**Authors:** Charles Hodge


### JAMES W. ALEXANDER, D.D.

BY CHARLES HODGE, D. D.

He never occupied a post which he did not adorn, and may well be pronounced "*blessed.*" He died in the full maturity of his years, and at the culminating point of his usefulness, leaving behind him *no superior*, and few, if any, equals in the sphere in which God had called him to act. His health recently becoming feeble he repaired to the mountains of his native State. Every thing promised a speedy recovery, but an acute disease (dysentery) prevailing in that vicinity, soon disappointed all our hopes. Early on Sabbath morning last, just before the sun began to throw his radiance over the land, he heard the Saviour say, "Come, ye blessed of my Father," &c.

When such men die, we are tempted almost to despair. It seems impossible that their place should be filled, or that we could ever do without them. Dr. Alexander united excellencies and gifts rarely found in one person—great intellect, refinement of taste, musical ear, &c. There was no more accomplished scholar. He was familiar with the Latin, Greek, and Hebrew, the French, German, Spanish and Italian languages—was more like Macaulay in the fullness of his style than any living writer. Many of his works are like strings of pearls—each a separate gem, yet bound to each other by an invisible thread. He once said that the only trouble he found in writing, was *in turning the leaves of his manuscript.* He was an erudite theologian, well acquainted with Christian doctrine in all its phases. These, however, were accomplishments; underneath these adornments was *the man*, and the *Christian*—"an Israelite indeed in whom there was no guile." Perhaps there is no man living more free from malice, from envy, from ill-will, and so abounding in things true and just and lovely and of good report. He was preëminently a devout man, fearing God, and full of the Holy Ghost.

Remarkable vivacity and versatility characterized his preaching. He preached Christ in a manner almost peculiar. He endeavored to turn men from themselves, and persuade them to accept a salvation wrought out for them. He was eminently successful not only in the conversion of sinners, but in guiding inquirers, and leading the people of God to higher attainments in piety. But the great charm of his preaching was his power over the religious affections; calling up joy, gratitude and love. His prayers were all *real acts* of adoration, thanksgiving and supplication. All his services were truly seasons of devotion, and inspired the very highest form of enjoyment ever vouchsafed to man on earth.

The man who can raise our hearts and bring us into communion with our Saviour, we cannot but reverence and love. It is a power which attracts all eyes, wins all hearts, and offends no one. Not any *one* thing, but the combination of all these, made not the first orator, but the first pastor in the land. To sit under his ministrations year after year, was a privilege to be coveted.

He was a man of great sorrow. When he entered Heaven, angels might say, "This is one that has come out of great tribulation, who has washed his robes and made them white in the blood of the Lamb." No man can *fill* his place. He was blessed with pious ancestors, but how supremely blessed *now*, since he has finished his course, having kept the faith, and received the crown.

In view of such a life and such a death, all the distinctions of earth sink into insignificance. Who would not rather be such a minister and such a servant of God, than the greatest warrior and conqueror the world ever knew? The great lesson taught by such a life and such a death is, that Christ is the Alpha and the Omega, the beginning and the end, the first and the last. He will say eventually to all his believing people, "Come, ye beloved."

